# Antibacterial activity and mechanism of plant flavonoids to gram-positive bacteria predicted from their lipophilicities

**DOI:** 10.1038/s41598-021-90035-7

**Published:** 2021-05-18

**Authors:** Ganjun Yuan, Yingying Guan, Houqin Yi, Shan Lai, Yifei Sun, Seng Cao

**Affiliations:** 1 Laboratory of Natural Medicine and Microbiological Drug, College of Bioscience and Bioengineering, Jiangxi Agricultural University, Nanchang, 330045 China; 2 Biotechnological Engineering Center for Pharmaceutical Research and Development, Jiangxi Agricultural University, Nanchang, 330045 China

**Keywords:** Secondary metabolism, Natural products, Antimicrobials, Natural products

## Abstract

Antimicrobial resistance seriously threatened human health, and new antimicrobial agents are desperately needed. As one of the largest classes of plant secondary metabolite, flavonoids can be widely found in various parts of the plant, and their antibacterial activities have been increasingly paid attention to. Based on the physicochemical parameters and antibacterial activities of sixty-six flavonoids reported, two regression equations between their ACD/LogP or LogD_7.40_ and their minimum inhibitory concentrations (MICs) to gram-positive bacteria were established with the correlation coefficients above 0.93, and then were verified by another sixty-eight flavonoids reported. From these two equations, the MICs of most flavonoids against gram-positive bacteria could be roughly calculated from their ACD/LogP or LogD_7.40_, and the minimum MIC was predicted as approximately 10.2 or 4.8 μM, more likely falls into the range from 2.6 to 10.2 μM, or from 1.2 to 4.8 μM. Simultaneously, both tendentiously concave regression curves indicated that the lipophilicity is a key factor for flavonoids against gram-positive bacteria. Combined with the literature analyses, the results also suggested that the cell membrane is the main site of flavonoids acting on gram-positive bacteria, and which likely involves the damage of phospholipid bilayers, the inhibition of the respiratory chain or the ATP synthesis, or some others.

## Introduction

Antimicrobial resistance (AMR) has been seriously threatened human public health and global economic development, and new antimicrobial agents are desperately needed^1,2^. Antibiotics, as the secondary metabolites produced by many bacteria, actinomycetes and fungi, showed remarkably antimicrobial activities, while they also bring some toxic side effects to human body, and are unavoidable to lead to the resistance^3^. Many plant ingredients present weaker antimicrobial activities, while some of them can reverse the resistance of antimicrobial agents^4^. Simultaneously, most of them are considered nontoxic to human body because of their ubiquity in all sorts of plant derived foods and beverages.

As one of the largest classes of plant secondary metabolite, flavonoids can be widely found in various parts of the plants, such as fruit, vegetables, nuts and tea^4^. These compounds have a wide range of pharmacological activities including antibiosis, antioxidation, and coronary heart disease prevention, etc. It is worth noting that some flavonoids can enhance the sensitivity of bacteria to antibiotics, and even reverse the AMR^4,5^. Thereout, the antibacterial activities of flavonoids have been paid more and more attention to. Recently, several investigations were performed for the antimicrobial activities of flavonoids, and the probable relationships between their chemical structures and antimicrobial activities were also summarized^4–6^. However, the regularity conclusions on the structure–activity relationships of flavonoids against bacteria still need to be further explored.

During our researches on antimicrobial agents^7–9^, it is vaguely found that the antimicrobial activities of flavonoids are not related to their special structure, while may be related to their polarities or lipid-water partition coefficients. Many data of plant flavonoids, involving their chemical structures and antibacterial activities reported in previous papers, were searched and analyzed for proving it. The inhibitory activities of plant flavonoids against gram-positive bacteria especially *Staphylococcus aureus* can be widely searched, while those against gram-negative ones and fungi were reported too few to carry out statistical analyses^4,6^. Thereby, the former was our focus in this research. As the inhibitory activities of a compound against different pathogenic bacteria are varied, this paper will pay more attention to the inhibitory activities of these flavonoids against *Staphylococcus aureus*, a species most reported in the literature.

## Results

### Structure, antibacterial activity, and physicochemical parameter

**Figure 1:**
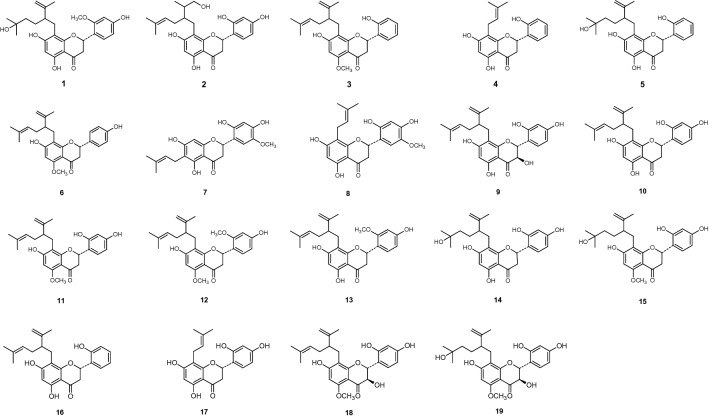
Chemical structures of compounds **1** to **19**^10^.

**Figure 2:**
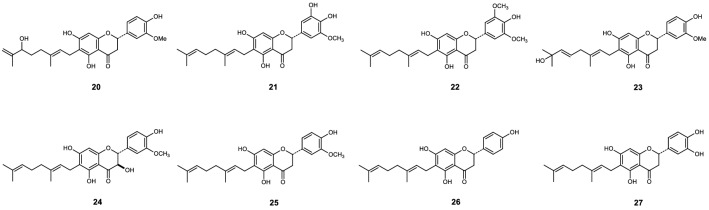
Chemical structures of compounds **20** to **27**^11^.

**Figure 3:**
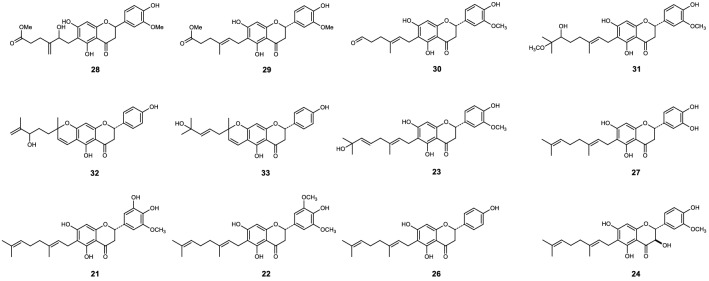
Chemical structures of compounds **21** to **24**, and **26** to **33**^12^.

**Figure 4:**
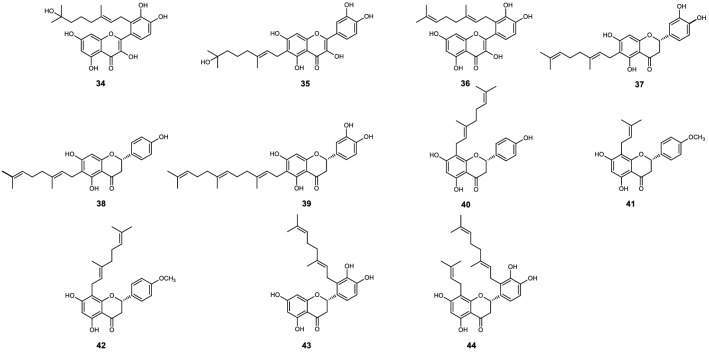
Chemical structures of compounds **34** to **44**^13^.

**Figure 5:**
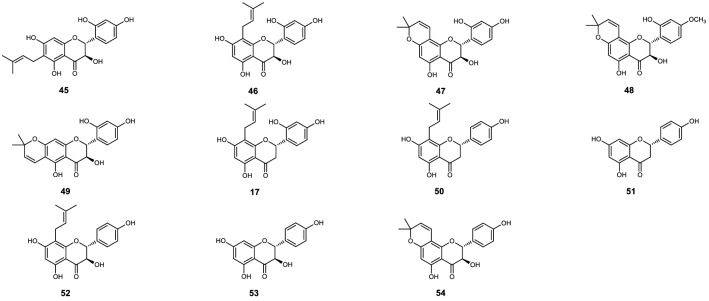
Chemical structures of compounds **17**, and **45** to **54**^14^.

**Figure 6:**
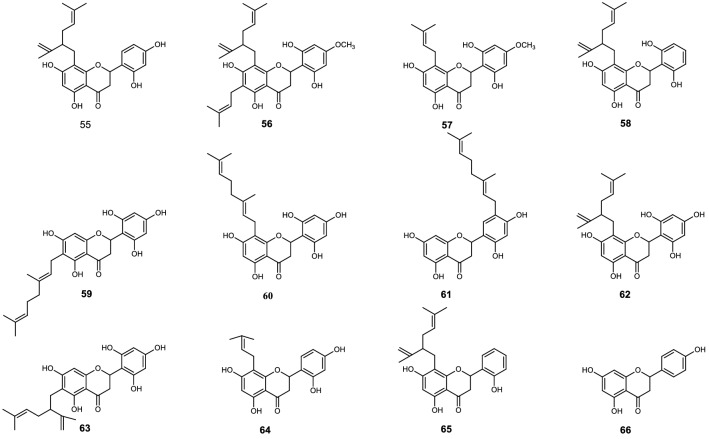
Chemical structures of compounds **55** to **66**^15^.

Sixty-six flavonoids (**1** to **66**) shown on Figs. 1, 2, 3, 4, 5 and 6, reported in six papers^10–15^, were selected for the preliminary structure-physicochemical parameter-activity analyses of plant flavonoids against gram-positive bacteria, especially *Staphylococcus aureus*. These flavonoids include three subclasses as flavonols, dihydroflavones and dihydroflavonols. Regression analyses indicated that no universal correlation between the antimicrobial activity (expressed as minimum inhibitory concentration, MIC) and the physicochemical parameter Gibbs energy, LogP (Partition coefficient), MR (Molar Refractivity), CMR (Calculated Molar Refractivity), tPSA (Topological Polar Surface Area), or solubility (SolDB) could be established for these flavonoids. However, probable correlations between the antimicrobial activities (MIC, or MIC_90_ which expressed as the MIC of a compound to 90% test isolates of a specific pathogen) and the physicochemical parameter CLogP (Calculated Partition coefficient), ACD/LogP, or LogD_7.40_ (Log_10_ of distribution coefficient at pH 7.40) were respectively discovered, and the physicochemical parameters and antimicrobial activities of these compounds were listed in Tables 1, 2, 3, 4, 5 and 6 for further analyses^10–15^.

**Table 1:** Physico-chemical parameters and antimicrobial activities of compounds **1** to **19**^10^.

Compounds	CLogP^a^	ACD/LogP^b^	LogD_7.40_^b^	MIC (μM)^c^	
				*S. aureus*	*B. subtilis*
**1**	4.67	5.55	5.38	L	L
**2**	4.08	5.09	4.92	11.3	11.3
**3**	6.31	7.02	6.80	11.8	5.9
**4**	4.35	5.29	5.09	14.7	14.7
**5**	4.52	5.52	5.35	–	–
**6**	6.36	7.02	6.81	23.7	23.7
**7**	3.53	4.18	4.09	25.9	25.9
**8**	3.58	4.18	3.98	25.9	25.9
**9**	4.50	5.74	5.50	22.7	22.7
**10**	5.58	6.52	6.33	5.9	5.9
**11**	5.64	6.30	6.08	5.7	5.7
**12**	6.46	7.05	6.83	5.5	5.5
**13**	6.40	7.27	7.09	5.7	5.7
**14**	3.86	4.80	4.63	L	L
**15**	3.92	4.58	4.37	L	L
**16**	6.25	7.24	7.06	12.2	6.1
**17**	3.68	4.56	4.37	14.0	7.0
**18**	4.57	5.53	5.26	–	–
**19**	2.84	3.81	3.56	–	–

^a^The CLogP values were calculated using software ChemBioDraw Ultra 12.0.

^b^The ACD/Log P and LogD_7.40_ values were calculated using software ACD/Labs 6.0.

^c^MIC, minimum inhibitory concentration; *S. aureus*, *Staphylococcus aureus*; *B. subtilis*, *Bacillus subtilis*; L, lower activity than other compounds while no data was given; –, no data was given.

**Table 2:** Physico-chemical parameters and antimicrobial activities of compounds **20** to **27**^11^.

Compounds	CLogP^a^	ACD/LogP^b^	LogD_7.40_^b^	MIC (μM)^c^	
				*S. aureus*	*S. epidermidis*
**20**	4.22	5.56	5.34	35.2	70.4
**21**	5.68	6.54	6.32	4.4	8.8
**22**	6.01	6.61	6.39	8.5	8.5
**23**	4.22	5.18	4.96	140.8^d^	140.8^d^
**24**	5.15	6.25	5.97	4.4	4.4
**25**	6.23	7.02	6.81	18.2	9.1
**26**	6.38	7.32	7.12	19.6	9.8
**27**	5.78	6.72	6.51	9.4	9.4

^a^The CLogP values were calculated using software ChemBioDraw Ultra 12.0.

^b^The ACD/Log P and LogD_7.40_ values were calculated using software ACD/Labs 6.0.

^c^MIC, minimum inhibitory concentration; *S. aureus*, *Staphylococcus aureus* ATCC 25923; *S. epidermidis*, *Staphylococcus epidermidis* ATCC 12228.

^d^Both MICs of compound **23** against *S. aureus* ATCC 25923 and *S. epidermidis* ATCC 12228 were more than 32 μg/mL (70.4 μM). As microdilution broth method was used to test MIC, we set 64 μg/mL (140.8 μM) as their MICs.

**Table 3:** Physico-chemical parameters and antimicrobial activities of compounds **21** to **24**, and **26** to **33**^12^.

Compounds	CLogP^a^	ACD/LogP^b^	LogD_7.40_^b^	MIC (μM)^c^		
				MRSA 6975	MRSA 630	MRSA 6205
**28**	1.83	3.27	3.04	140.2	280.4^d^	280.4^d^
**29**	3.68	4.60	4.38	36.2	144.6	72.3
**30**	3.28	4.27	4.05	19.4	155.2	77.6
**31**	3.91	4.67	4.46	32.9	263.2	263.2
**32**	4.47	6.10	5.76	18.9	151.5	151.5
**33**	4.47	5.63	5.29	37.9	151.5	151.5
**23**	4.22	5.18	4.96	17.6	35.2	35.2
**27**	5.78	6.72	6.51	18.8	37.7	37.7
**21**	5.68	6.54	6.32	17.6	8.8	8.8
**22**	6.01	6.61	6.39	17.1	17.1	8.5
**26**	6.38	7.32	7.12	4.9	9.8	4.9
**24**	5.15	6.25	5.97	8.8	8.8	17.6

^a^The CLogP values were calculated using software ChemBioDraw Ultra 12.0.

^b^The ACD/Log P and LogD_7.40_ values were calculated using software ACD/Labs 6.0.

^c^MIC, minimum inhibitory concentration; MRSA 6975, methicillin-resistant *Staphylococcus aureus* 6975; MRSA 630, methicillin-resistant *Staphylococcus aureus* 630; MRSA 6205, methicillin-resistant *Staphylococcus aureus* 6205.

^d^The MICs of compound **28** against MRSA 630 and 6205 were more than 64 μg/mL (140.2 μM). As microdilution broth method was used to test MIC and the three physico-chemical parameters were small, we set 128 μg/mL (280.4 μM) as their MICs.

**Table 4:** Physico-chemical parameters and antimicrobial activities of compounds **34** to **44**^13^.

Compounds	CLogP^a^	ACD/LogP^b^	LogD_7.40_^b^	MIC (μM)^c^	
				*S. aureus*	*B. subtilis*
**34**	3.46	4.52	3.84	140.2	140.2
**35**	3.71	4.52	3.93	140.2	140.2
**36**	5.18	6.20	5.53	73.0	73.0
**37**	5.78	6.72	6.51	9.5	9.5
**38**	6.38	7.32	7.12	19.7	9.8
**39**	7.81	8.75	8.54	32.6	16.3
**40**	6.43	7.32	7.13	9.8	39.4
**41**	4.98	5.94	5.75	90.8	90.8
**42**	7.01	7.97	7.78	19.0	19.0
**43**	5.78	6.74	6.5	37.9	37.9
**44**	7.73	8.84	8.64	8.2	16.3

^a^The CLogP values were calculated using software ChemBioDraw Ultra 12.0.

^b^The ACD/Log P and LogD_7.40_ values were calculated using software ACD/Labs 6.0.

^c^MIC, minimum inhibitory concentration; *S. aureus*, *Staphylococcus aureus* 209P; *B. subtilis*, *Bacillus subtilis* NBRC 3134.

**Table 5:** Physico-chemical parameters and antimicrobial activities of compounds **17**, and **45** to **54**^14^.

Compounds	CLogP^a^	ACD/LogP^b^	LogD_7.40_^b^	MIC_90_ (μM)^c^	
				MRSA (22)	MSSA (7)
**45**	2.55	3.79	3.67	167.8	335.7
**46**	2.60	3.79	3.53	167.8	167.8
**47**	2.65	3.92	3.59	42.1	42.1
**48**	3.42	4.67	4.35	81.4	40.6
**49**	2.65	4.11	3.67	84.5	84.5
**17**	3.68	4.56	4.37	175.4	350.8
**50**	4.40	5.29	5.10	183.6	183.6
**51**	2.44	3.19	2.96	> 918.3	> 918.3
**52**	3.32	4.51	4.27	87.8	87.8
**53**	1.37	2.42	2.11	1734.6^d^	1734.6^d^
**54**	3.37	4.64	4.34	88.3	88.3

^a^The CLogP values were calculated using software ChemBioDraw Ultra 12.0.

^b^The ACD/Log P and LogD_7.40_ values were calculated using software ACD/Labs 6.0.

^c^MIC_90_, minimum inhibitory concentration to 90% test isolates; MRSA (22), twenty-two isolates of methicillin-resistant *Staphylococcus aureus*; MSSA (7), seven isolates of methicillin-susceptible *Staphylococcus aureus.*

^d^Both MIC_90_s of compound **53** against MRSA and MSSA were more than 250 μg/mL (867.3 μM). As microdilution broth method was used to test MIC, we set 500 μg/mL (1734.6 μM) as their MIC_90_s.

**Table 6:** Physico-chemical parameters and antimicrobial activities of compounds **55** to **66**^15^.

Compounds	CLogP^a^	ACD/LogP^b^	LogD_7.40_^b^	MIC (μM)^c^	
				MRSA G31	MRSA G47
**55**	5.58	6.52	6.33	14.7	7.4
**56**	7.58	8.76	8.70	12.0	6.0
**57**	3.78	4.72	4.51	16.2	32.3
**58**	5.53	6.52	6.33	14.7	14.7
**59**	4.94	5.89	5.67	28.4	7.1
**60**	4.99	5.89	5.68	28.4	14.2
**61**	5.71	6.60	6.35	29.4	14.7
**62**	4.86	5.81	5.62	28.4	28.4
**63**	4.81	5.81	5.62	28.4	28.4
**64**	3.68	4.56	4.37	35.1	35.1
**65**	6.25	7.24	7.06	122.4^d^	122.4^d^
**66**	2.44	3.19	2.96	1469.2	734.6

^a^The CLogP values were calculated using software ChemBioDraw Ultra 12.0.

^b^The ACD/Log P and LogD_7.40_ values were calculated using software ACD/Labs 6.0.

^c^MIC, minimum inhibitory concentration; MRSA G31 and G47, methicillin-resistant *Staphylococcus aureus* G31 and G47.

^d^Both MICs of compound **65** against MRSA G31 and G47 were more than 25 μg/mL (61.2 μM). As microdilution broth method was used to test MIC, we set 50 μg/mL (122.4 μM) as their MICs.

### Data analysis and correlation establishment

The regression analyses for the physicochemical parameters CLogP, ACD/LogP, or LogD_7.40_ and the antimicrobial activities (MIC or MIC_90_) of these flavonoids to a certain pathogenic bacterium were respectively performed, and their regression curves were showed on Fig. S1 to S6 in Supplementary Information. From these figures, nearly all regression curves indicate that the antibacterial activities of these flavonoids present similar change characteristics along with the increase of their LogP or LogD_7.40_. First, the antibacterial activities will dramatically increase when the LogP or LogD_7.40_ increase up to a specific value. Along with the further increase of LogP or LogD_7.40_, the antibacterial activities will first increase tendentiously and then decrease. Simultaneously, their regression equations between the physicochemical parameter (*x*) and the MIC (*y*), together with the correlation coefficients (*r*), were respectively presented on Fig. S1 to S6, and summarily listed in Table 7. Most correlation coefficients (*r*) were more than 0.90 (Table 7). This indicated that there is a good correlation between the physicochemical parameter CLogP, ACD/LogP, or LogD_7.40_ and the antimicrobial activities (MIC), of these flavonoids to a certain pathogenic bacterium.

**Table 7:** Regression equations between the physicochemical parameter (*x*) and the antimicrobial activity (*y*) to a certain pathogenic microorganism.

Compounds	Parameters^b^	Pathogenic bacteria^a^	Regression equation (*r*^c^)
**1** to **19**	CLogP	*S. aureus*	*y* = −14.562*x*^5^ + 368.41*x*^4^ − 3689.3*x*^3^ + 18274*x*^2^ − 44755*x* + 43,369 (0.8514)
	ACD/LogP		*y* = −6.1684*x*^5^ + 180.3*x*^4^ − 2090*x*^3^ + 12006*x*^2^ − 34172*x* + 38,560 (0.7592)
	LogD_7.40_		*y* = −5.3777*x*^5^ + 151.88*x*^4^ − 1700.4*x*^3^ + 9430.9*x*^2^ − 25910*x* + 28,225 (0.7331)
	CLogP	*B. subtilis*	*y* = −13.392*x*^5^ + 345.58*x*^4^ − 3527.6*x*^3^ + 17792*x*^2^ − 44315*x* + 43,606 (0.8093)
	ACD/LogP		*y* = −8.1245*x*^5^ + 238.87*x*^4^ − 2785.6*x*^3^ + 16098*x*^2^ − 46081*x* + 52,265 (0.8168)
	LogD_7.40_		*y* = −6.8012*x*^5^ + 193.67*x*^4^ − 2186.6*x*^3^ + 12230*x*^2^ − 33875*x* + 37,175 (0.7660)
**20** to **27**	CLogP	*S. aureus*	*y* = 8.524*x*^4^ − 204.37*x*^3^ + 1846.7*x*^2^ − 7432.5*x* + 11,221 (0.7878)
	ACD/LogP		*y* = −35.117*x*^5^ + 1129.5*x*^4^ − 14526*x*^3^ + 93407*x*^2^ − 300407*x* + 386,677 (0.9998)
	LogD_7.40_		*y* = −32.854*x*^5^ + 1023.1*x*^4^ − 12742*x*^3^ + 79351*x*^2^ − 247194*x* + 308,250 (0.9998)
	CLogP	*S. epidermidis*	*y* = 27.806*x*^4^ − 635.4*x*^3^ + 5428.8*x*^2^ − 20549*x* + 29,078 (0.9228)
	ACD/LogP		*y* = 49.336*x*^5^ − 1560.6*x*^4^ + 19638*x*^3^ − 122806*x*^2^ + 381390x − 470,183 (0.9999)
	LogD_7.40_		*y* = 50.433*x*^5^ − 1541.1*x*^4^ + 18726*x*^3^ − 113020*x*^2^ + 338580*x* − 402,381 (0.9999)
**21** to **24**, and **26** to **33**	CLogP	MRSA 6975	*y* = 1.8495*x*^4^ − 35.986*x*^3^ + 255.94*x*^2^ − 793.24*x* + 933.27 (0.9727)
	ACD/LogP		*y* = 4.3462*x*^4^ − 100.91*x*^3^ + 865.04*x*^2^ − 3250.5*x* + 4550 (0.9737)
	LogD_7.40_		*y* = 3.9824*x*^4^ − 89.302*x*^3^ + 738.93*x*^2^ − 2679.6*x* + 3624.8 (0.9724)
	CLogP	MRSA 630	*y* = 6.6617*x*^4^ − 108.73*x*^3^ + 631.7*x*^2^ − 1592.5*x* + 1668.4 (0.8594)
	ACD/LogP		*y* = 6.7373*x*^4^ − 144.65*x*^3^ + 1138.9*x*^2^ − 3954.4*x* + 5320.3 (0.8317)
	LogD_7.40_		*y* = 0.7482*x*^5^ − 12.599*x*^4^ + 58.825*x*^3^ + 49.926*x*^2^ − 1007.8*x* + 2110.7 (0.8409)
	CLogP	MRSA 6205	*y* = 13.675*x*^4^ − 236.81*x*^3^ + 1465.8*x*^2^ − 3839.6*x* + 3692.3 (0.8157)
	ACD/LogP		*y* = −1.087*x*^5^ + 45.877*x*^4^ − 674.86*x*^3^ + 4547.7*x*^2^ − 14384*x* + 17,445 (0.7738)
	LogD_7.40_		*y* = −5.6301*x*^5^ + 162.46*x*^4^ − 1831.6*x*^3^ + 10059*x*^2^ − 26887*x* + 28,095 (0.7847)
**34** to **44**	CLogP	*S. aureus*	*y* = 5.0053*x*^3^ − 76.039*x*^2^ + 329.45*x* − 293.29 (0.9720)
	ACD/LogP		*y* = 4.1508*x*^3^ − 74.07*x*^2^ + 387.42*x* − 479.73 (0.9651)
	LogD_7.40_		*y* = 3.2426*x*^3^ − 54.386*x*^2^ + 256.74*x* − 225.91 (0.9643)
	CLogP	*B. subtilis*	*y* = 3.2197*x*^3^ − 46.825*x*^2^ + 177.07*x* − 40.407 (0.9622)
	ACD/LogP		*y* = 3.0215*x*^3^ − 52.494*x*^2^ + 254.63*x* − 216.12 (0.9619)
	LogD_7.40_		*y* = 2.3606*x*^3^ − 38.598*x*^2^ + 166.87*x* − 63.384 (0.9607)
**17**, **45** to **54**	CLogP	MRSA	*y* = −187.27*x*^3^ + 2012.3*x*^2^ − 7014.5*x* + 8045.6 (0.9982)
	ACD/LogP		*y* = −141.22*x*^3^ + 2038.8*x*^2^ − 9673*x* + 15,205 (0.9972)
	LogD_7.40_		*y* = −138.02*x*^3^ + 1870.5*x*^2^ − 8315.2*x* + 12,249 (0.9964)
	CLogP	MSSA	*y* = −186.51*x*^3^ + 1996*x*^2^ − 6936.4*x* + 7973.6 (0.9830)
	ACD/LogP		*y* = −129.02*x*^3^ + 1872.9*x*^2^ − 8962.9*x* + 14,287 (0.9789)
	LogD_7.40_		*y* = −146.22*x*^3^ + 1942.5*x*^2^ − 8495.7*x* + 12,386 (0.9760)
**55** to **66**	CLogP	MRSA G31	*y* = −17.547*x*^5^ + 454.87*x*^4^ − 4632.7*x*^3^ + 23200*x*^2^ − 57189*x* + 55,596 (0.9999)
	ACD/LogP		*y* = −12.103*x*^5^ + 371.48*x*^4^ − 4493.1*x*^3^ + 26798*x*^2^ − 78893*x* + 91,819 (0.9999)
	LogD_7.40_		*y* = −10.79*x*^5^ + 322.43*x*^4^ − 3789.8*x*^3^ + 21934*x*^2^ − 62584*x* + 70,524 (0.9999)
	CLogP	MRSA G47	*y* = −14.185*x*^5^ + 357.83*x*^4^ − 3530.1*x*^3^ + 17044*x*^2^ − 40327*x* + 37,493 (0.9997)
	ACD/LogP		*y* = −9.8483*x*^5^ + 294.98*x*^4^ − 3468.7*x*^3^ + 20038*x*^2^ − 56931*x* + 63,738 (0.9997)
	LogD_7.40_		*y* = −8.6059*x*^5^ + 250.58*x*^4^ − 2858.5*x*^3^ + 15993*x*^2^ − 43945*x* + 47,542 (0.9996)

^a^The antimicrobial activity (*y*) was expressed as MIC or MIC_90_ to a certain pathogenic microorganism. *S. aureus*, *Staphylococcus aureus*; *S. epidermidis*, *Staphylococcus epidermidis*; *B. subtilis*, *Bacillus subtilis*; MRSA, methicillin-resistant *Staphylococcus aureus*; MSSA methicillin-susceptible *Staphylococcus aureus.*

^b^CLogP was calculated using software ChemBioDraw Ultra 12.0, and ACD/LogP and LogD_7.40_ were calculated using software ACD/Labs 6.0.

^c^*r*, correlation coefficients.

**Figure 7:**
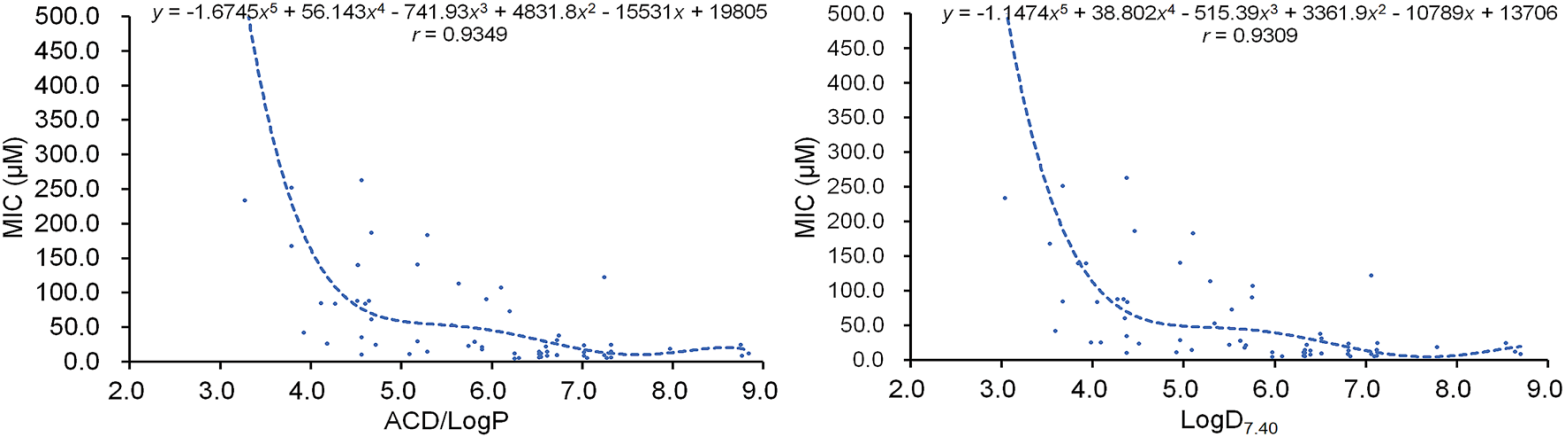
Polynomial regression analyses for the physicochemical parameters ACD/LogP or LogD_7.40_ (*x*) and the average MICs (*y*) to gram-positive bacteria including *S. aureus*, *S. epidermidis*, or/and *B. subtilis*, of compounds **1** to **66**.

As we pointed out above, the antimicrobial activities of a compound against different pathogenic bacteria were varied, and even against the same one in different determination conditions. Thereby, the regression analyses were respectively performed for these flavonoids reported in different papers. Considering that the pathogenic bacteria used for antibacterial experiments mainly involved *S. aureus*, *S. epidermidis*, and *B. subtilis*, the same compound should present similar inhibitory activities and identical antibacterial mechanism to these gram-positive bacteria. Thereby, we put the physicochemical parameters and the average MICs to *S. aureus*, *S. epidermidis*, or/and *B. subtilis* (Tables 1, 2, 3, 4, 5 and 6), of these flavonoids together for further regression analyses. The results indicated that the correlation between CLogP and antibacterial activities (MICs) is weak with a correlation coefficient of 0.8412, while that between ACD/LogP or LogD_7.40_ (*x*) and MICs (*y*) is more reliable (Fig. 7). The regression equations were respectively expressed as *y* = − 1.6745*x*^5^ + 56.143*x*^4^ − 741.93*x*^3^ + 4831.8*x*^2^ − 15531*x* + 19,805 and *y* = − 1.1474*x*^5^ + 38.802*x*^4^ − 515.39*x*^3^ + 3361.9*x*^2^ − 10789*x* + 13,706, with the correlation coefficients of 0.9349 and 0.9309, respectively. These further proved, by a larger sample, that the inhibitory activities of these flavonoids to gram-positive bacteria will nonlinearly increase as the ACD/LogP or LogD_7.40_ increase to approximately 7.0, and then decrease along with the further increase of ACD/LogP or LogD_7.40_.

### Verification

**Figure 8:**
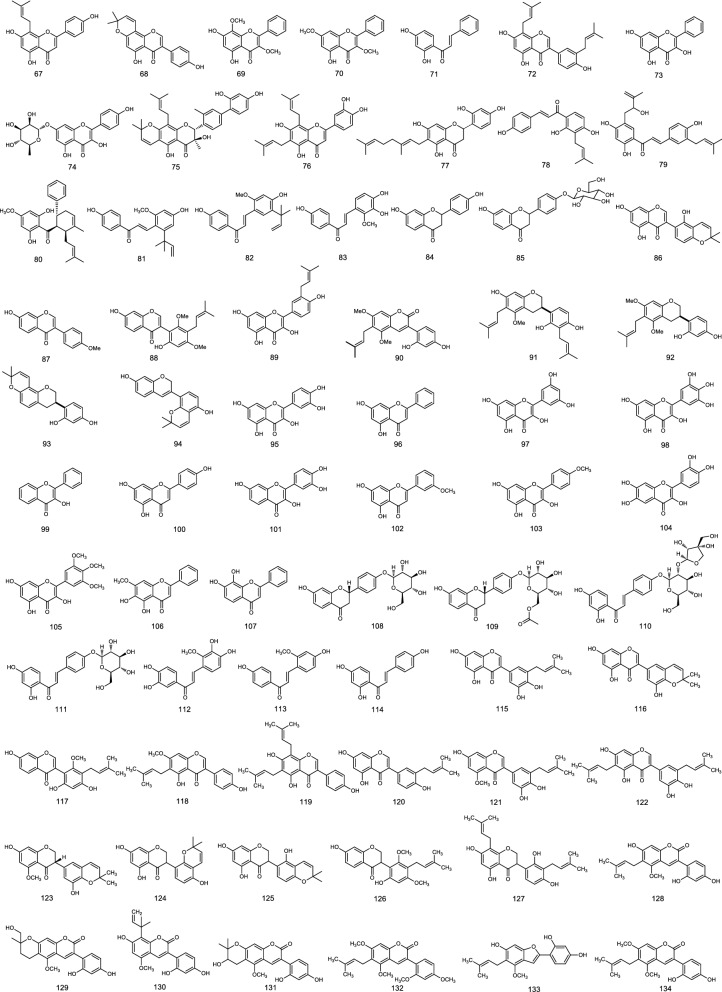
Chemical structures of compounds **67** to **134**^4,16–21^.

To verify the above correlations, other sixty-eight flavonoids (Fig. 8) including flavone, isoflavone, flavonol, flavanonol, dihydroflavone, dihydroisoflavone, flavane, and chalcone subclasses etc., reported in seven papers^4,16–21^, were selected for the comparison of theoretical and reported MICs. Using above two regression equations *y* = − 1.6745*x*^5^ + 56.143*x*^4^ − 741.93*x*^3^ + 4831.8*x*^2^ − 15531*x* + 19,805 and *y* = − 1.1474*x*^5^ + 38.802*x*^4^ − 515.39*x*^3^ + 3361.9*x*^2^ − 10789*x* + 13,706 (*x* is the ACD/LogP or LogD_7.40_, and *y* is the antimicrobial activities (MICs)), the theoretical MICs of these flavonoids can be calculated. Considering that many factors, such as determination method, concentration of bacterial suspension, and test medium used, may influence on the determination of MIC^5^, the results reported would fluctuate within a reasonable range of the actual values. Thereout, the predicted MICs ranged from 1/4 × to 4 × the determined one were acceptable (marked as A), especially those ranged from 1/2 × to 2 × the determined one, were considered as complete coincidence (marked as C) since the MICs were generally determined by double dilution method^22^. Simultaneously, those more than or equal to the minimum value when the determined MICs were no upper limit were also regarded as complete coincidence (marked as C). Otherwise, those were unacceptable (marked as U). The results (Table 8) indicated that the predicted MICs were in acceptable or complete coincidence with the measured ones for approximate 85.3% flavonoids. Although the antibacterial activities of ten flavonoids (14.7%) are unsatisfactorily predicted, there are six compounds with the predicted MICs falling into the range of 1/8 × to 8 × determined ones. This together indicated that the MICs of most flavonoids against gram-positive bacteria can be roughly calculated from their ACD/LogP or LogD_7.40_ although the predicted values are not in accordance with their tested ones for a few flavonoids. At least, these indicated that the ACD/LogP or LogD_7.40_ is a key factor for the inhibitory activities of plant flavonoids against gram-positive bacteria.

**Table 8:** Comparison of predicted and reported antibacterial activities of some flavonoids.

Compounds	Molecular weight	Predicted antibacterial activities^a^				Measured antibacterial activities		Coincidence^b^	Reference
		By LogP		By LogD_7.40_					
		LogP	MIC (μM)	LogD_7.40_	MIC (μM)	MIC (μg/mL)	MIC (μM)		
**67**	338.35	4.20	121.21	3.77	162.30	62.5	184.7	C	16
**68**	336.34	5.55	54.21	4.82	52.69	62.5	185.8	A	16
**69**	314.29	2.16	2467.72	1.72	2794.56	≥ 200	≥ 636.4	C	17
**70**	298.29	3.10	695.19	2.32	1381.83	≥ 200	≥ 670.5	C	17
**71**	240.25	3.93	183.65	3.62	206.48	50–200	208.1–832.5	C	17
**72**	406.47	7.33	14.22	6.89	19.22	1.56–3.13	3.8–7.7	C	4
**73**	270.24	2.83	1030.88	2.16	1683.78	> 125–240	> 462.6–888.1	C	4
**74**	432.38	1.70	4166.24	0.76	7234.60	1–2	2.3–4.6	U	4
**75**	542.62	8.63	23.00	8.17	13.80	2.3–37.5	4.4–69.1	C	4
**76**	422.47	6.59	31.55	6.40	32.27	0.5–4	1.2–9.5	A	4
**77**	424.49	6.60	31.25	6.42	31.74	2.9	6.8	U	4
**78**	324.37	5.49	54.80	5.44	47.91	0.3–0.6	0.9–1.9	U	4
**79**	408.49	5.95	48.36	5.70	45.79	0.6–1.22	1.5–3.0	U	4
**80**	406.51	8.35	21.21	7.93	10.00	0.125–2	0.3–4.9	A	4
**81**	338.4	4.95	61.35	4.82	52.69	3.13–16	9.3–47.3	C	4
**82**	338.4	4.95	61.35	4.82	52.69	3.13–6.25	9.4–18.9	A	18,19
**83**	286.28	2.57	1471.41	2.44	1185.40	> 25–100	> 87.3–349.3	C	18,19
**84**	256.25	2.76	1137.07	2.61	946.52	> 100	> 390.2	C	18,19
**85**	418.39	0.61	11,968.2	0.46	9405.99	> 50	> 119.5	C	18,19
**86**	352.34	5.67	52.85	5.07	49.83	12.5	35.5	C	18,19
**87**	268.26	3.15,16	644.51	2.91	621.60	> 25–100	> 93.2–372.8	C	18,19
**88**	382.41	5.38	55.79	5.12	49.51	> 25–50	> 65.4–130.8	C	18,19
**89**	354.35	4.15,16	130.39	3.48	258.70	12.5–25	35.3–70.6	C	18,19
**90**	382.41	5.75	51.77	5.75	45.21	> 50	> 130.8	U	18,19
**91**	424.53	6.32	39.42	6.32	34.31	3.13–6.25	7.4–14.7	A	18,19
**92**	370.44	4.41	93.10	4.40	68.92	6.25–12.5	16.9–33.7	A	18,19
**93**	324.37	4.18	124.77	4.18	88.60	6.25–12.5	19.3–38.5	A	18,19
**94**	322.35	6.64	30.07	6.63	26.09	12.5–25	38.8–77.6	C	18,19
**95**	302.24	2.07	2746.05	1.40	3919.38	> 125	> 413.6	C	20
**96**	254.24	2.88	960.17	2.33	1364.52	> 125	> 491.7	C	20
**97**	302.24	2.62	1376.51	1.95	2158.21	500	1654.3	C	20
**98**	318.24	2.11	2619.40	1.42	3839.98	62.5–125	196.4–392.8	U	20
**99**	238.24	3.76	242.43	3.74	170.26	> 125	> 524.7	U	20
**100**	270.24	2.1	2650.61	1.57	3284.32	≥ 125	≥ 462.6	C	20
**101**	286.24	2.52	1571.57	2.22	1564.90	> 125	> 436.7	C	20
**102**	284.26	3.04	760.45	2.50	1096.06	> 125	> 439.7	C	20
**103**	300.26	3.00	806.77	2.33	1364.52	> 125	> 416.3	C	20
**104**	302.24	2.54	1530.86	2.14	1724.98	31.3–62.5	103.4–206.8	U	20
**105**	360.31	3.02	783.32	2.32	1381.83	> 125	> 346.9	C	20
**106**	284.26	3.22	578.89	2.45	1170.12	> 125	> 439.7	C	20
**107**	254.24	2.51	1592.25	2.19	1623.47	31.3–62.5	122.9–245.8	U	20
**108**	418.39	0.61	11,968.2	0.46	9405.99	> 128	> 305.9	C	21
**109**	460.43	2.46	1699.00	2.31	1399.32	> 128	> 278.0	C	21
**110**	550.51	2.24	2239.77	2.06	1897.96	> 128	> 232.5	C	21
**111**	418.39	1.04	8107.91	0.87	6546.46	> 128	> 305.9	C	21
**112**	302.28	2.36	1929.91	2.19	1623.47	> 128	> 423.5	C	21
**113**	270.28	3.23	570.00	3.10	468.93	64–128	236.8–473.6	C	21
**114**	256.25	3.40	436.17	3.26	366.65	128	499.5	C	21
**115**	354.35	5.03	59.77	4.48	64.08	32–64	90.3–180.6	C	21
**116**	352.34	4.63	73.82	4.07	102.86	32–64	90.8–181.6	C	21
**117**	368.38	5.33	56.22	5.08	49.76	64	173.73	A	21
**118**	352.38	5.69	52.59	5.40	48.13	16	45.41	C	21
**119**	406.47	7.33	14.22	7.16	13.23	8	19.68	C	21
**120**	338.35	5.24	57.06	4.69	55.65	16–32	47.3–94.6	C	21
**121**	368.38	4.70	69.97	4.27	79.27	64	173.7	A	21
**122**	422.47	7.13	17.41	6.89	19.22	16	37.9	C	21
**123**	368.38	4.56	78.51	4.27	79.27	32–64	86.9–173.7	C	21
**124**	354.35	5.47	54.98	5.21	49.04	32–64	90.3–180.6	C	21
**125**	354.35	5.47	54.98	5.21	49.04	32	90.3	C	21
**126**	384.42	4.83	64.65	4.67	56.23	32–64	83.2–166.5	C	21
**127**	424.49	6.69	28.60	6.50	29.62	8	18.9	C	21
**128**	368.38	5.99	47.55	5.98	41.74	16	43.4	C	21
**129**	384.38	3.76	242.43	3.76	164.91	≥ 128	≥ 333.0	C	21
**130**	368.38	5.61	53.56	5.59	46.85	16–32	43.4–86.9	C	21
**131**	384.38	3.86	205.75	3.86	140.83	> 128	> 333.0	C	21
**132**	410.46	5.79	51.17	5.79	44.71	> 128	> 311.9	U	21
**133**	340.37	4.10	140.56	4.10	98.63	16	47.0	A	21
**134**	382.41	5.75	51.77	5.75	45.21	> 50–128	> 130.8–334.7	A	21

Antibacterial activities were expressed as MICs of flavonoids to gram-positive bacteria which include *S. aureus*, *S. epidermidis* and *B. subtilis.*

^a^LogP and LogD_7.40_ were calculated using software ACD/Labs 6.0.

^b^C, Complete coincidence; A, Acceptable; U, Unacceptable.

## Discussion and conclusion

Flavonoids can be widely found in various parts of the plant, and their antibacterial activities have been paid more and more attention to, especially after some of them were discovered to have the potency to enhance the susceptibility of some antibiotics to bacteria^4,5^. Based on the related data of plant flavonoids reported, many related physicochemical parameters were calculated, using software ChemBioDraw Ultra 12.0 and ACD/Labs 6.0, for the discovery of the correlations between the physicochemical parameters and the MICs of flavonoids against gram-positive bacteria. Two regression equations between the ACD/LogP or LogD_7.40_ (*x*) and the antimicrobial activities (MICs) (*y*) were established as *y* = − 1.6745*x*^5^ + 56.143*x*^4^ − 741.93*x*^3^ + 4831.8*x*^2^ − 15531*x* + 19,805 and *y* =  − 1.1474*x*^5^ + 38.802*x*^4^ − 515.39*x*^3^ + 3361.9*x*^2^ − 10789*x* + 13,706. From these two equations, the MICs of most flavonoids against gram-positive bacteria (mainly Staphylococcus and Bacillus) could be roughly calculated from their ACD/LogP or LogD_7.40_, and their minimum value was predicted as approximately 10.2 or 4.8 μM. Considering that the experimental MICs would fluctuate within a reasonable range^5^, the minimum MIC of plant flavonoids will likely fall into the range from 2.6 to 10.2 μM, or from 1.2 to 4.8 μM, predicted from their ACD/LogP or LogD_7.40_.

After all, the antibacterial activities of a compound to different pathogens are varied, and so these two regression equations, mainly valuable for Staphylococcus and Bacillus, may not always be suitable for flavonoids to other gram-positive bacteria. However, the acceptable range from 1/4 × to 4 × the determined MICs will increase the applicability of these two equations used for the prediction of plant flavonoids to other gram-positive bacteria. To say the least, if necessary, similar regression equations can be also established from the physicochemical parameters and the MICs to other gram-positive bacteria, of flavonoids. Thereby, we concluded that the MICs of most flavonoids against gram-positive bacteria can be roughly calculated from their physicochemical parameters ACD/LogP or LogD7.40.

Lipophilicity is a very important descriptor indicating membrane permeation^23^, and generally expressed as LogP which is valid only for a single electrical species. For ionizable drugs, LogD that refers to a pH-dependent mixture of all electrical species presented at any given pH was regarded as a better descriptor reflecting the actual partitioning and lipophilicity^24,25^. Generally, most flavonoids contain two or more phenolic hydroxyl groups^4–6^, and present similar weak acidity with the pKa of 7.0 to 10.0. Thereby, their LogD will correspondingly decrease along with the increase of environmental pH from about 5.0. Considering the pH in human blood or in the media of MIC determination was approximately 7.40, their LogD at pH 7.40 were selected. These together above indicate that the lipophilicity of plant flavonoids is a key factor for their inhibitory activities to gram-positive bacteria. As the lipophilicity is closely related to membrane permeability^26^, the tendentiously concave regression curves between the antibacterial activity and the LogP or LogD_7.40_ also indicate that the cell membrane is probably an important site of flavonoids acting on gram-positive bacteria.

Different antibacterial mechanisms of plant flavonoids were reported^4–6^, such as causing cell-membrane damage, inhibition on various synthase involving the nucleic acid synthesis, the bacterial respiratory chain, or the cell envelope synthesis. However, the results above suggested that the antibacterial activities of these plant flavonoids had no obvious relationship with the specific fragments of their structures, while presented great relationship with their lipophilicities. Simultaneously, the antibacterial activities of plant flavonoids will dramatically increase as the LogP or LogD increases from 2.5 to 4.0 which range the membrane permeability remarkably decrease while the affinity to lipid bilayer greatly increase^27–29^. According to this, plant flavonoids may not target specific synthases, but more likely to nonspecifically act on the cell-membrane bilayer or the respiratory chain to kill bacteria. This deduction was indirectly supported by many researches which were reviewed in three paper^4–6^, such as follows: (1) two mechanisms may be involved the interactions of flavonoids with lipid bilayers, which include the interactions at the membrane interface between the polar heads of phospholipids and the more hydrophilic flavonoids, and the partition of the more hydrophobic flavonoids in the interior of the lipid bilayer^30^; (2) nonspecific interactions of flavonoids with phospholipids can lead to the changes of the membrane properties^31^; (3) The increased activities of more lipophilic flavonoids are due to the enhanced membrane affinity of their long acyl chains^32^; (4) Some lipophilic flavonoids can decrease the fluidity and integrity of cellular membrane to inhibit gram-positive bacteria^33,34^, such as sophoraflavanone G and 3-arylideneflavanones.

Although many other antibacterial mechanisms acting on various synthase for the nucleic acid or cell envelope syntheses were mentioned in these reviews^4,6^, two facts found from the researches of the cited literature are worth further discussing. First, most flavonoids used for mechanism exploration have the cLogP ranged from about 2.0 to 4.0, and are easy to infiltrate into the bacterial cell, while they present very weak antibacterial activities with the MICs more than 250 μg/mL. Second, most experiments were achieved by the determination of enzyme activities *in vitro*^35,36^, the molecular docking of flavonoids with various synthases^37^, the proteomics technology without the combination of related experiments and the consideration of first the chicken or the egg^38^. Another thing should be considered is whether some molecules can pass through the cell membrane and infiltrate into the bacterial cell or not. Moreover, previous works indicated the antibacterial activity to gram-positive bacteria was observed only four of fourteen flavonoids, while only four of seven flavonoids with DNA gyrase inhibition showed weak inhibitory activity to gram-positive bacteria^20^. Simultaneously, the authors pointed out that mechanisms other than DNA gyrase inhibition may also play a role in the antibacterial activity. Thereby, the conclusion that some of these flavonoids studied are potent inhibitors of DNA gyrase is worth reconsidering^20^. In fact, this work just right indicated that the inhibitory activity of flavonoids against gram-positive bacteria did not correlate with their in vitro DNA gyrase inhibition to a large extent. This was also supported by previous publication^39^. These together further confirmed that the cell-membrane should be the main region of plant flavonoids acting on Gram-positive bacteria, and which likely involving the disruption or damage of phospholipid bilayers, the inhibition of the respiratory chain or ATP synthesis, or some others.

According to the regression equations and above conclusions, the inhibitory activities of flavonoids to gram-positive bacteria will increase when the alkyl especially isopentyl were introduced into the structures of flavonoids no matter carbon position it is introduced into. This can be interpreted that the introduction of alkyl would increase the lipophilicity of flavonoids or the LogP, and thereout increase their interactions with phospholipids of cell membrane. However, the introduction of too many alkyls will overmuch increase the LogP of these flavonoids, and which will lead their lipophilicities too large to pass through the hydrophilic region of phospholipid bilayers. This was proved by previous similar work^26,32,40^. On the contrary, the inhibitory activities of flavonoids to gram-positive bacteria will decrease when polar groups, such as hydroxyl and glycosyl, were introduced into their structures. This can be interpreted as that the excessive hydrophilicity of flavonoids will hinder its infiltration into phospholipid bilayers and interaction with hydrophobic region of cell membrane.

Based on the physicochemical parameters and MICs of various flavonoids, the regression equations and above conclusions were achieved. For a certain subclass of flavonoids, the regression equations with larger correlation coefficient can be established for their more accurate MIC predictions, and then can be further used for the structural design and optimization to obtain more efficient antibacterial activity.

As the inhibitory activities of plant flavonoids against gram-negative bacteria were reported less, it is difficult to draw a statistical conclusion. Considering that the cell envelope of gram-negative bacteria was different from that of gram-positive ones, it is worth further exploring whether the above regression equations and above conclusions are suitable for plant flavonoids against gram-negative bacteria. However, these can provide good references for their related researches. Referring to the above conclusions, the anti-MRSA activities of trimethylhydroquinone, vitamin K_3_ and carnosic acid were successfully predicted and verified by our laboratory^9,41^.

**Figure 9:**
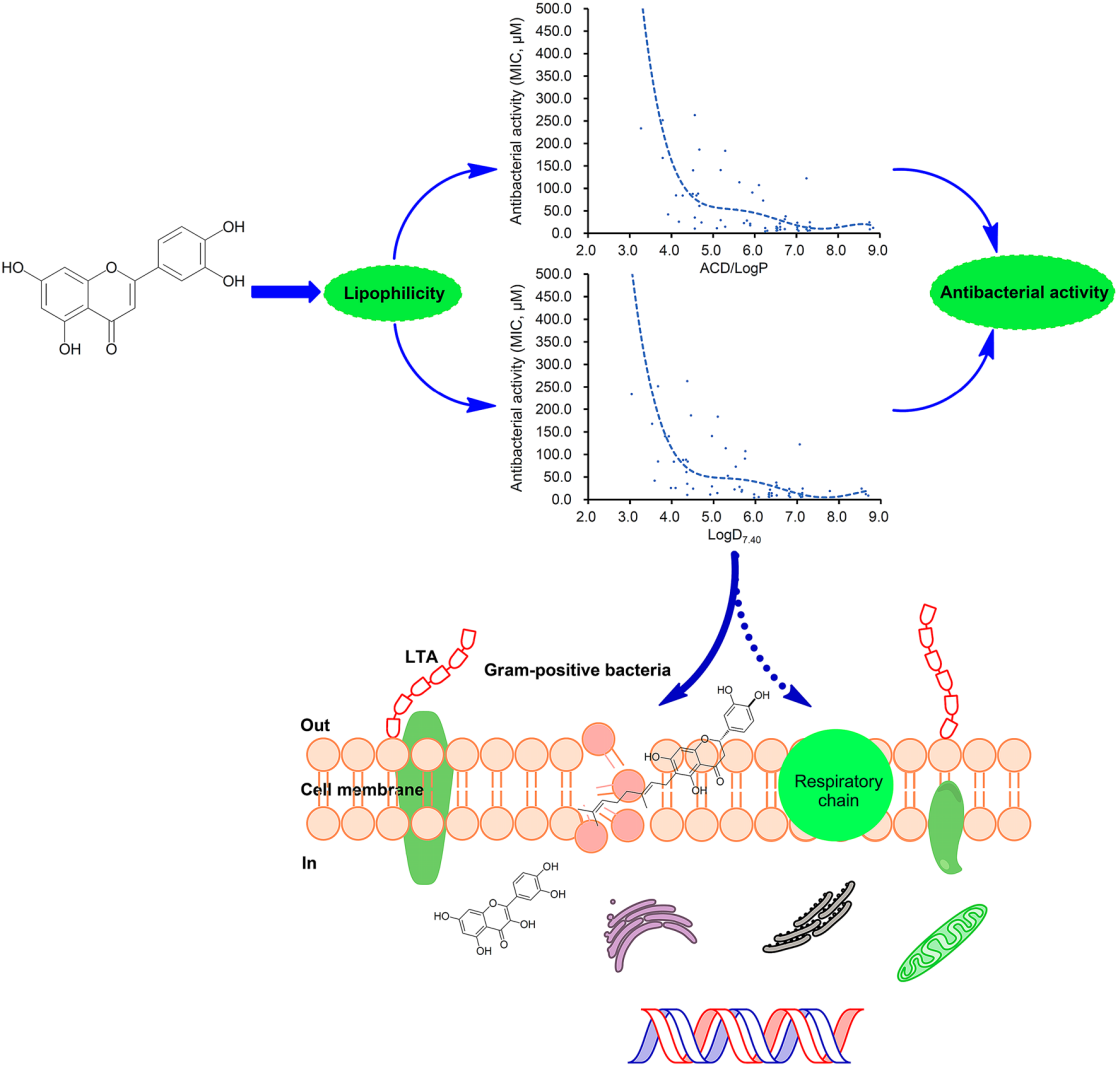
Diagrammatic presentation for the inhibitory activities and mechanisms of plant flavonoids to gram-positive bacteria.

In conclusion, the MICs of most flavonoids against gram-positive bacteria can be roughly calculated from their physicochemical parameters ACD/LogP or LogD_7.40_, and the lipophilicity is a key factor of plant flavonoids against gram-positive bacteria. Combined with the analyses of previous publications, the results also suggest that the cell membrane may be the main site of plant flavonoids acting on gram-positive bacteria, and which likely involves the damage of phospholipid bilayers, the inhibition of the respiratory chain or ATP synthesis, or some others. Base on this, the inhibitory activities and mechanisms of plant flavonoids to gram-positive bacteria were diagrammatically presented as Fig. 9.

## Methods

### Information and data

The structures, antimicrobial activities and other related information of plant flavonoids were unsystematically searched from Google academic search engine, and several databases SciFinder, Medline, Elsevier, ACS, ScienceDirect, Wiley Online Library, Springer-Link, and RSC, using keywords flavonoid and antimicrobial, or and antibacterial, and or and anti-MRSA. Furthermore, the relevant references in the obtained literature were also tracked. The structures, antibacterial activities, and other related information of flavonoids were collected from the obtained literature that can provide more than five or more flavonoids. As the antimicrobial activities of a certain compound against different pathogenic strains were varied, compounds reported in different papers were independently collected for the following analyses. Finally, the structures of selected compounds were drawn using software ChemBioDraw Ultra 12.0.

### Simulation calculation of physicochemical parameters

The physicochemical parameters Gibbs energy, LogP, CLogP, MR, CMR and tPSA were calculated using software ChemBioDraw Ultra 12.0. Moreover, another software ACD/Labs 6.0 was also used for the calculations of physicochemical parameters LogP, LogD_7.40_ and solubility (SolDB).

### Data analysis and correlation establishment

The physicochemical parameters and antibacterial activities of flavonoids reported in the same paper were respectively listed in a table, even those of the same compound. The regression analyses between the calculated values of each parameter and the antimicrobial activities (expressed as MICs) of all compounds in a table were respectively performed using Microsoft Excel software. It is noting that compounds without related antimicrobial information were not considered for the regression analyses, while they can be used for the following discussion. The physicochemical parameters significantly correlating with the antimicrobial activities were selected for the further analyses of correlations between the physicochemical parameters and antimicrobial activities of flavonoids.

### Verification

Some other flavonoids were searched from above several databases, and the chemical structures of various flavonoids presented in previous publications were also drawn using software ChemBioDraw Ultra 12.0. The physicochemical parameters LogP and LogD_7.40_ of these flavonoids were respectively calculated by software ACD/Labs 6.0, and then their antimicrobial activities (MICs) were respectively predicted using the above regression equations. Comparing with the predicted MICs with the determined one, the regression equations can be verified.

## Supplementary Information

Supplementary Figures.

## Data Availability

The datasets generated during and/or analyzed during the current study are available from the corresponding author on reasonable request.
