# Supplementary material for: Antibacterial activity and mechanism of plant flavonoids to gram-positive bacteria predicted from their lipophilicities
Source: Sci Rep. 2021 May 18;11:10471. doi: 10.1038/s41598-021-90035-7 (PMC8131645; doi:10.1038/s41598-021-90035-7)
Supplement: Supplementary file 1 — Supplementary Figures. [file 41598_2021_90035_MOESM1_ESM.pdf]

Supplementary Information

Antibacterial Activity and mechanism of plant flavonoids to gram-positive bacteria predicted from their lipophilicities

Ganjun Yuan[1,2,*], Yingying Guan[1], Houqin Yi[1], Shan Lai[1,2], Yifei Sun[1], Seng Cao[1,2]

[1]Laboratory of Natural Medicine and Microbiological Drug, College of Bioscience and Bioengineering, Jiangxi Agricultural University, Nanchang 330045, China

[2]Biotechnological Engineering Center for Pharmaceutical Research and Development, Jiangxi Agricultural University, Nanchang 330045, China

[*]Correspondence: Ganjun Yuan, gyuan@jxau.edu.cn

Figures S1 to S6

Fig. S1 Polynomial regression analyses for the physicochemical parameters CLogP, ACD/LogP or $LogD_{7.40}$ (x) and the MIC (y) to *Staphylococcus aureus* (left line) or *Bacillus subtilis* (right line), of compounds **1** to **19**[10].

Fig. S2 Polynomial regression analyses for the physicochemical parameters CLogP, ACD/LogP or $LogD_{7.40}$ (x) and the MIC (y) to *S. aureus* ATCC 25923 (left line) or *S. epidermidis* ATCC 12228 (right line), of compounds **20** to **27**[11].

Fig. S3 Polynomial regression analyses for the physicochemical parameters CLogP, ACD/LogP or $LogD_{7.40}$ (x) and the MIC (y) to methicillin-resistant *Staphylococcus aureus* (MRSA) 6975 (upper row), MRSA 630 (middle row) or MRSA 6205 (lower row), of compounds **21** to **24**, and **26** to **33**[12].

Fig. S4 Polynomial regression analyses for the physicochemical parameters CLogP, ACD/LogP or $LogD_{7.40}$ (x) and the MIC (y) to MRSA 209P (left line) or *B. subtilis* NBRC 3134 (right line), of compounds **34** to **44**[13].

Fig. S5 Polynomial regression analyses for the physicochemical parameters CLogP, ACD/LogP or $LogD_{7.40}$ (x) and the MIC (y) to MRSA (left line) or methicillin-susceptible *S. aureus* (right line), of compounds **17**, **45** to **54**[14].

Fig. S6 Polynomial regression analyses for the physicochemical parameters CLogP, ACD/LogP or $LogD_{7.40}$ (x) and the MIC (y) to MRSA G31 (left line) or G47 (right line), of compounds **55** to **66**[15].

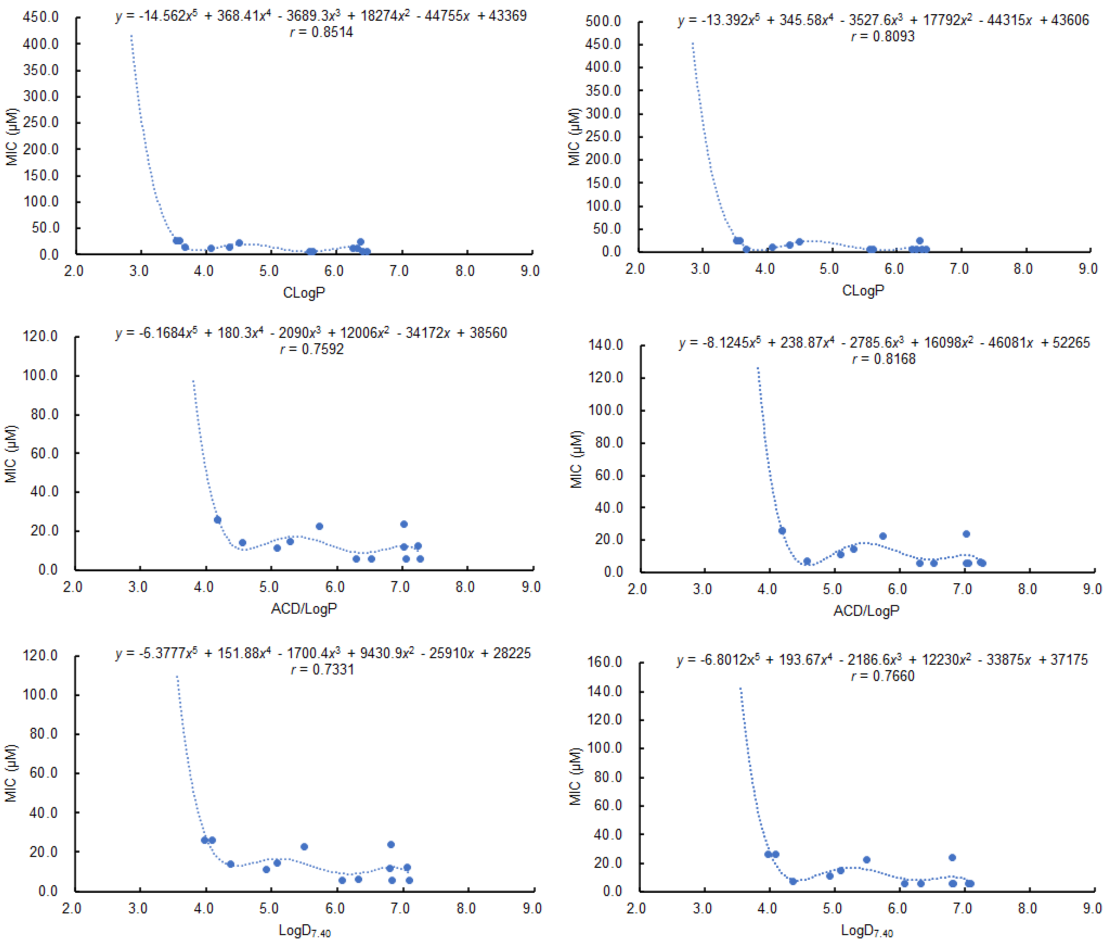

Fig. S1 Polynomial regression analyses for the physicochemical parameters CLogP, ACD/LogP or LogD$_{7.40}$ (x) and the MIC (y) to *Staphylococcus aureus* (left line) or *Bacillus subtilis* (right line), of compounds **1** to **19**[10].

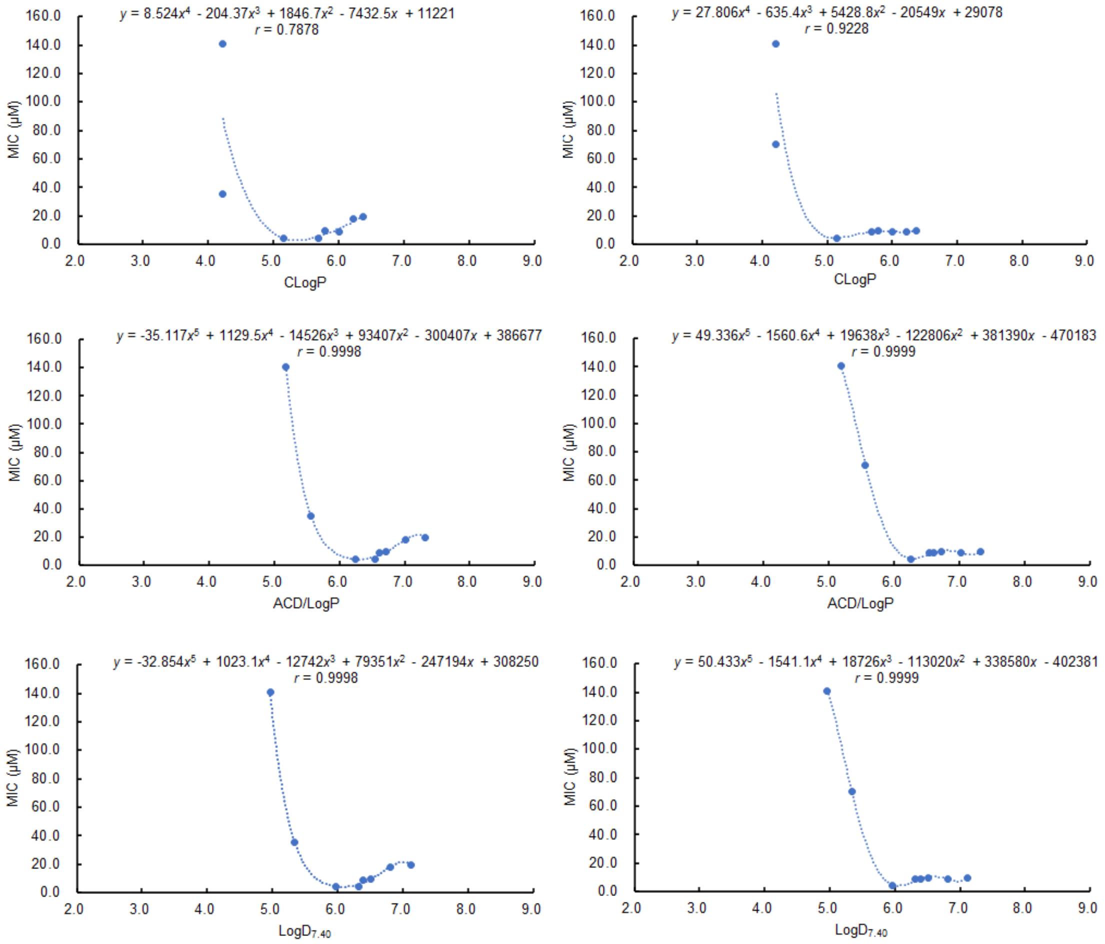

Fig. S2 Polynomial regression analyses for the physicochemical parameters CLogP, ACD/LogP or LogD$_{7.40}$ (*x*) and the MIC (*y*) to *S. aureus* ATCC 25923 (left line) or *S. epidermidis* ATCC 12228 (right line), of compounds **20** to **27**[11].

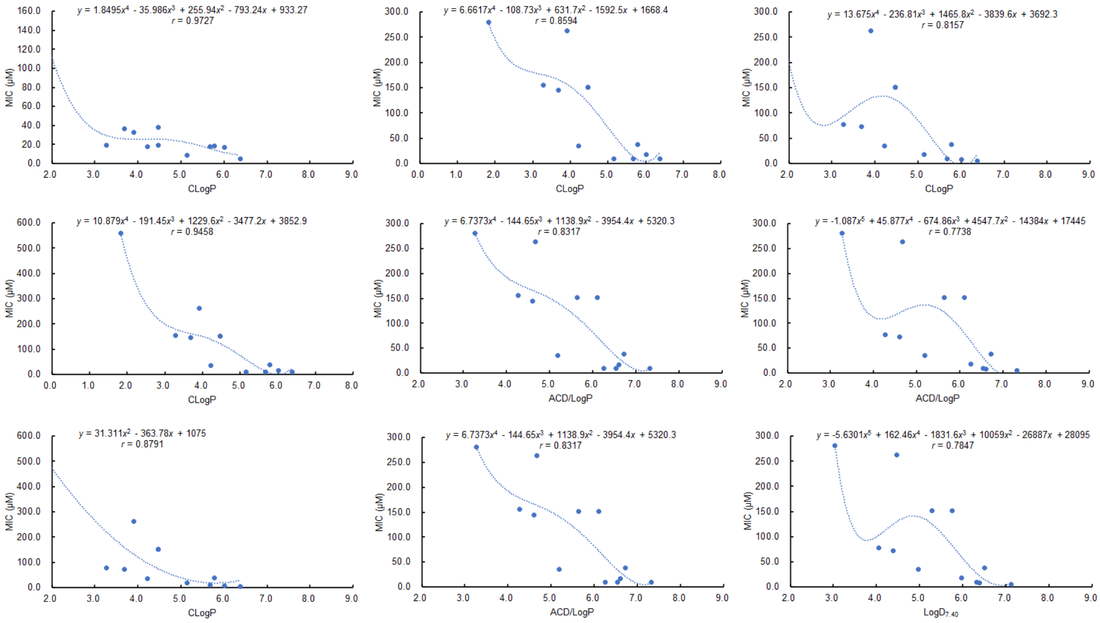

Fig. S3 Polynomial regression analyses for the physicochemical parameters CLogP, ACD/LogP or $LogD_{7.40}$ (x) and the MIC (y) to methicillin-resistant *Staphylococcus aureus* (MRSA) 6975 (upper row), MRSA 630 (middle row) or MRSA 6205 (lower row), of compounds **21** to **24**, and **26** to **33**[12].

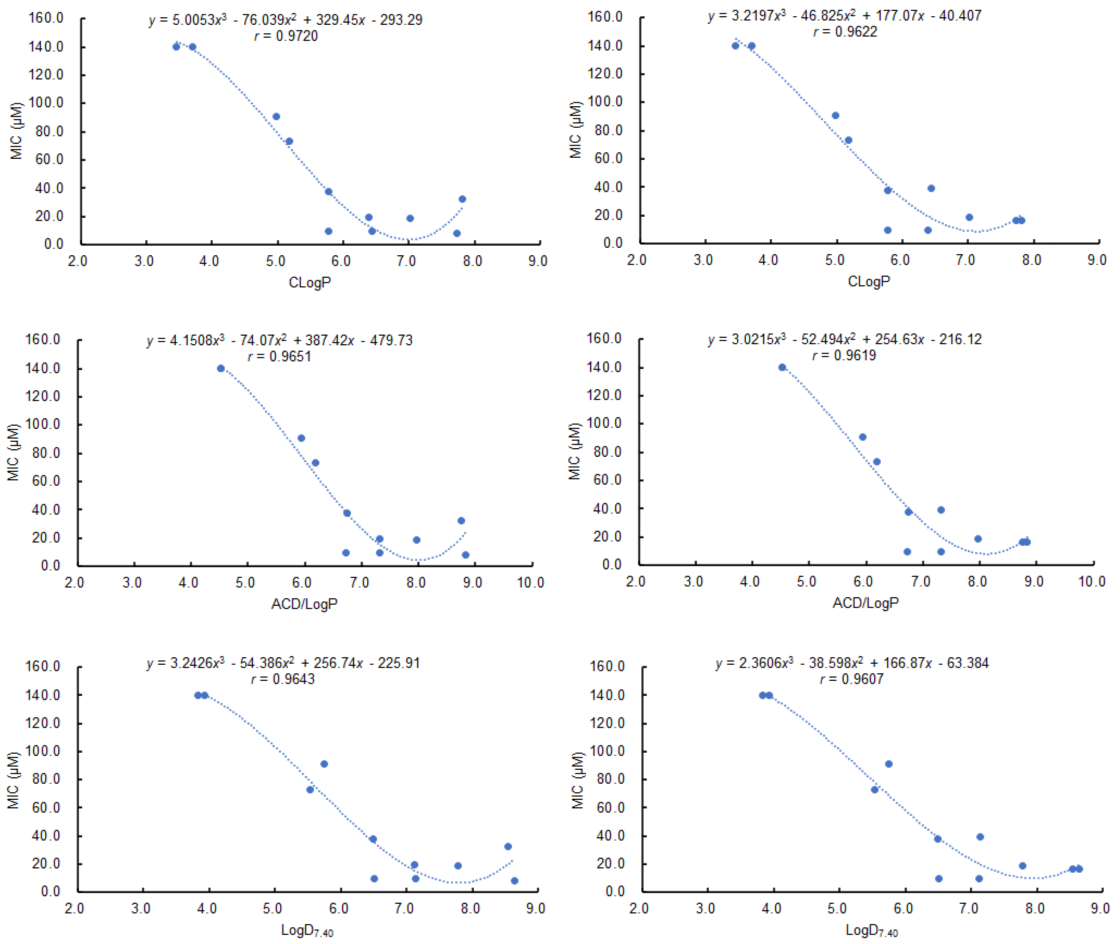

Fig. S4 Polynomial regression analyses for the physicochemical parameters CLogP, ACD/LogP or $LogD_{7.40}$ (x) and the MIC (y) to MRSA 209P (left line) or *B. subtilis* NBRC 3134 (right line), of compounds **34** to **44**[13].

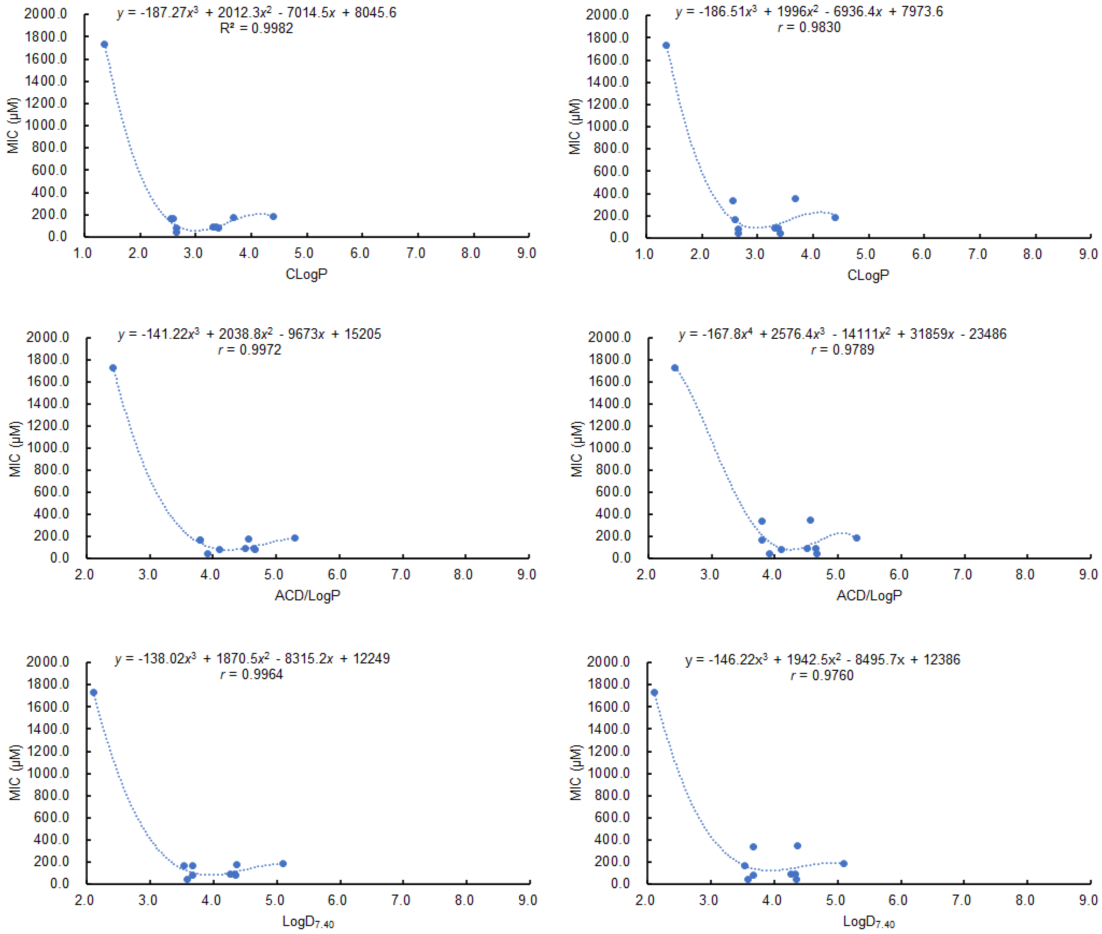

Fig. S5 Polynomial regression analyses for the physicochemical parameters CLogP, ACD/LogP or $LogD_{7.40}$ (x) and the MIC (y) to MRSA (left line) or methicillin-susceptible *S. aureus* (right line), of compounds **17**, **45** to **54**[14].

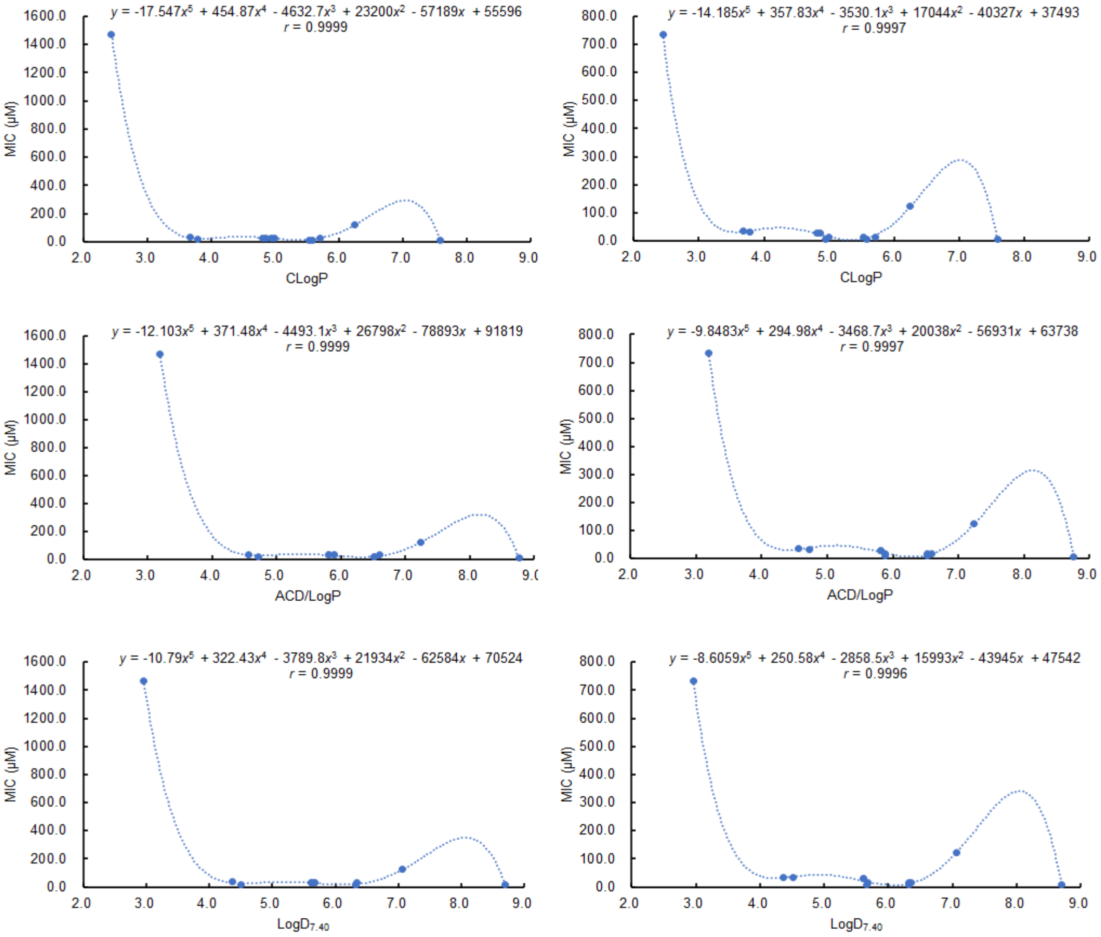

Fig. S6 Polynomial regression analyses for the physicochemical parameters CLogP, ACD/LogP or LogD$_{7.40}$ (x) and the MIC (y) to MRSA G31 (left line) or G47 (right line), of compounds **55** to **66**[15].

Acknowledgements

This work was supported by grants from the National Natural Science Foundation of China (No. 82073745, 81960636 and 81660578).

Author contributions

G.Y. initiated the project, hypothesis and design, analyzed the data and obtained regression equations, wrote the manuscript, took part in the search and collection of flavonoids data, prepared the figures and tables; Y.G. performed the search and collection of flavonoids data, took part in the preparation of figures and tables; H.Y., S.L., Y.S. and S.C. took part in the data processing and analysis. All authors reviewed the manuscript.

Additional Information

Competing Interests: The author declare that they have no competing interests.
